# A Scoping Review of the Clinical Utility of Laparoscopic Vasectomy

**DOI:** 10.7759/cureus.69022

**Published:** 2024-09-09

**Authors:** Khang Duy Ricky Le, Annie Jiao Wang, Shasha Haycock, Matthew J Shears, Mark Forbes

**Affiliations:** 1 Geelong Clinical School, Deakin University School of Medicine, Geelong, AUS; 2 Department of General Surgical Specialties, The Royal Melbourne Hospital, Melbourne, AUS; 3 Department of Surgery, Northeast Health Wangaratta, Wangaratta, AUS; 4 Faculty of Medicine, Dentistry, and Health Sciences, The University of Melbourne, Melbourne, AUS; 5 Department of Urology, Northeast Health Wangaratta, Wangaratta, AUS

**Keywords:** hernia repair, laparoscopic hernia repair, laparoscopic vasectomy, laparoscopy, vasectomy

## Abstract

Vasectomy is a common procedure performed for family planning. Traditionally, this has been via a scrotal approach. In contrast, laparoscopic vasectomy is a documented but rarely described procedure that may minimise anaesthetic risk, surgical risk, and healthcare expenditure in patients undergoing elective laparoscopic procedures for concurrent pathology such as hernia repair. This scoping review evaluates the clinical utility of laparoscopic vasectomy. It was performed in accordance with the Preferred Reporting Items for Systematic Reviews and Meta-Analyses extension for Scoping Reviews (PRISMA-ScR). Articles were identified with keywords related to laparoscopy and vasectomy. Six peer-reviewed, full-text articles published in English were included in this review. These studies encompass eight individual patient cases of laparoscopic vasectomy performed in the 1990s and early 2000s. All the cases included laparoscopy for concurrent pathology, the most common of which was inguinal hernia. There were no complications associated with laparoscopic vasectomy. For patients requiring laparoscopic surgery for alternate pathologies, synchronous laparoscopic vasectomy improves surgical efficiency by minimising anaesthetic time, operative time, and risk, in addition to lower associated healthcare costs. However, consideration is given to the limitations of this approach, and a note is made of the lack of evidence regarding safety and efficacy given the paucity of cases described in the literature.

## Introduction and background

The paradigm for vasectomy is a minimally-invasive scrotal approach where small incisions are made to the scrotum, at the central scrotal raphe or bilaterally on the lateral scrotum, prior to isolation and division of the vas deferens [1]. Vasectomy in this way is performed commonly, with excellent outcomes in terms of safety and efficacy [2]. The laparoscopic approach is another option for vasectomy that has been reported. Laparoscopic vasectomy has been well described in veterinarian medicine, due to the varied anatomy of different animals including dogs, elephants, and horses [3-5].

In humans, the procedure is exceedingly rare, with a significant paucity of evidence and experience in performing laparoscopic vasectomy. Understandably, as a standalone procedure, laparoscopic vasectomy is more time-consuming, resource-intensive (requiring laparoscopic instruments, longer anaesthetic and recovery time) and costly. With the traditional minimally-invasive vasectomy offering a solution to many of these barriers, there are surgeons who suggest avoiding laparoscopic vasectomy entirely [6]. However, there is evidence indicating that there may be a role for laparoscopic vasectomy, particularly for patients undergoing simultaneous laparoscopy for alternate pathology [7]. Furthermore, by approaching the vasectomy in this way, there is the potential to avoid local complications, such as scrotal haematoma, infection, epididymo-orchitis, and sperm granulomas [8]. Despite this potential, the evidence surrounding appropriate clinical pathways to undertake laparoscopic over traditional vasectomy, as well as outcomes related to laparoscopic vasectomy, remain poorly characterised. This scoping review seeks to evaluate the current literature surrounding the utility, safety, and efficacy of laparoscopic vasectomy to inform best-practice clinical pathways where this procedure may be considered for patients.

## Review

Methods

Search Strategy

A scoping review was conducted in adherence to the Preferred Reporting Items for Systematic Reviews and Meta-Analyses extension for Scoping Reviews (PRISMA-ScR) [9]. A computer-assisted search was performed on MEDLINE (Medical Literature Analysis and Retrieval System Online), Embase, and Cochrane Controlled Register of Trials (CENTRAL) databases and the World Health Organisation (WHO) International Clinical Trials Registry Platform on July 20, 2024. The search combined relevant medical subject headings (MeSH) terms and keywords related to laparoscopy and vasectomy. Additional articles were manually from reference lists of relevant articles.

Study Selection

Peer-reviewed full-text articles available in English that evaluated laparoscopic vasectomy in adults (age > 18 years) were considered for this review. Articles were excluded if they evaluated other vasectomy approaches (such as no-scalpel vasectomy, traditional open vasectomy), laparoscopic vasectomy in animals, or the role of laparoscopy for vasectomy reversal. 

Literature Screening

Screening by title and abstract was performed independently by two researchers (KL, AW). Articles that did not provide sufficient information proceeded to full-text analysis. The same two researchers (KL, AW) then independently performed a full-text analysis of studies for eligibility. Disagreement during the process was resolved by discussion and consensus.

Outcomes of Interest

Primary outcomes of interest for this scoping review were related to the safety, efficacy and clinically relevant indications of laparoscopic vasectomy.

Results

Literature Search

**Figure 1:**
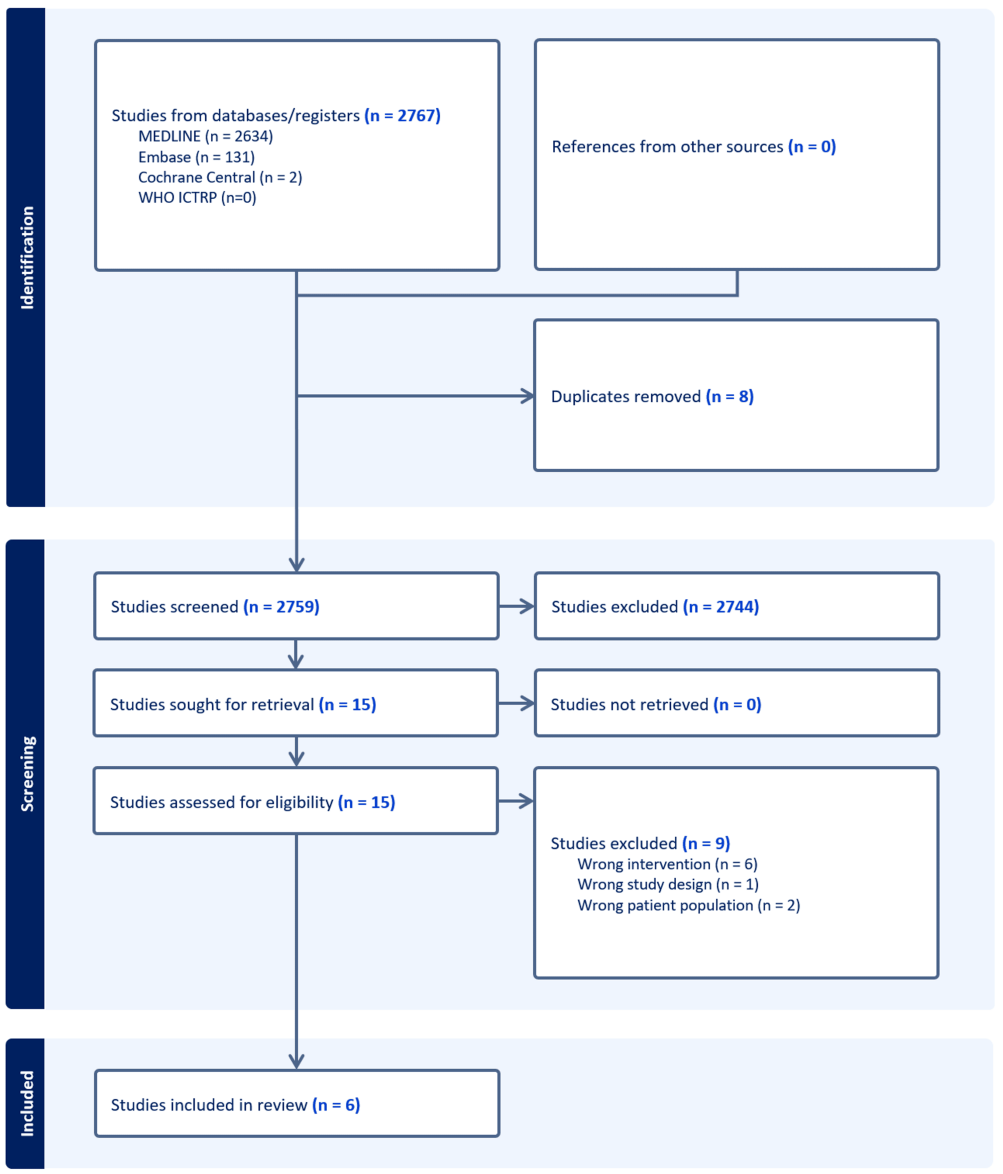
Flowchart of literature search and screening MEDLINE: Medical Literature Analysis and Retrieval System Online; ICTRP: International Clinical Trials Registry Platform

A total of 2767 articles were identified following a comprehensive literature search. After accounting for duplicates, 2759 articles were subsequently screened by title and abstract, with 15 articles progressing to full-text review. Of these, nine articles were excluded with six articles eligible for inclusion. The PRISMA flowchart of the literature search is shown in Figure 1.

Overview of Included Studies

An overview of included studies is presented in Table 1. A total of six articles were included, of which four were case reports, one was a letter to the editor describing a case from a single patient, and one was an editorial that described a case series of three patients [6,7,10-13]. Studies were published between the years 1992 and 2002. Four studies were from the United States [6,7,10,13], one from Canada [11], and one from Australia [12]. All studies reported on laparoscopic vasectomy. One study had insufficient data [13].

**Table 1:** Overview of included studies and patient characteristics NR: not reported

Study	Country of Publication	Study design	Sample size	Age	Indication for vasectomy	Concurrent pathology	Overview of treatment	Follow-up
Childers and Hicks, 1992 [10]	United States	Case report	1	38	Family planning	Unilateral cryptorchidism	38-year-old with unilateral intra-abdominal cryptorchidism and undesired fertility. Underwent laparoscopic orchiectomy and contralateral vasectomy.	Follow-up at 1 week was unremarkable for post-operative concerns.
Kakitelashvili et al., 2002 [7]	United States	Case report	1	42	Family planning	Bilateral inguinal hernia	42-year-old with unremarkable medical history. Underwent synchronous laparoscopic vasectomy performed at the time of a bilateral laparoscopic inguinal hernia repair with extraperitoneal approach. No complications	NR
Kasirajan et al., 1999 [6]	United States	Case report	1	48	Family planning	Right inguinal hernia	48-year-old with unremarkable medical history. Underwent laparoscopic right inguinal hernia repair with extraperitoneal approach with synchronous vasectomy.	Follow-up at 6 months demonstrated aspermia on semen analysis. No post-operative concerns.
Mosquera and Urban, 1994 [13]	United States	Case report	1	NR	NR	Hernia	Male of unclear age underwent laparoscopic repair of unidentified hernia with concurrent vasectomy.	NR
Patterson et al., 1996 [11]	Canada	Editorial	3	40s	Family planning	Two patients with abdominal wall hernias. One patient with chronic abdominal pain for investigation	Three patients in their 40s underwent laparoscopic vasectomy at the same time as laparoscopy for hernia repair (n=2) or investigation of chronic abdominal pain (n=1).	All patients were followed up at 1 month with no post-operative concerns. Semen analysis at 6 weeks demonstrated aspermia in all patients.
Smith and Polglase, 1993 [12]	Australia	Letter to the editor	1	36	Family planning	Right inguinal hernia	36-year-old with unremarkable medical history. Underwent laparoscopic right inguinal hernia repair with synchronous vasectomy.	NR

Patient Characteristics

The included studies encompass eight individual patients. Where complete data was available, all patients were in their late 30s or 40s. All patients had no significant comorbidities reported.

Indications for Laparoscopic Vasectomy

All patients with reportable data underwent laparoscopic vasectomy for desired infertility as part of family planning. All cases were described to have concurrent pathology that required laparoscopic management, which therefore allowed for synchronous laparoscopic vasectomy. Of the eight individual cases from the six articles, the most common concurrent pathology was the presence of either a unilateral or bilateral inguinal hernia (n = 5) which was managed with laparoscopic hernia repair. The remaining three individual cases were reported to have an unidentified hernia (n = 1), unilateral cryptorchidism requiring laparoscopic orchiectomy (n = 1), or chronic abdominal pain of unclear aetiology requiring diagnostic laparoscopy (n = 1). All cases pursued laparoscopic vasectomy to reduce the need for additional surgical wounds. There were no cases of standalone laparoscopic vasectomy.

Outcomes Following Laparoscopic Vasectomy

Variable follow-up was described, with data only available from three of the six included articles. Childers and Hicks reported no postoperative issues at the one-week follow-up; however, it is unclear whether aspermia was achieved at a later point [10]. Kasirajan et al. [6] and Patterson et al. [11] reported follow-up periods ranging from one to six months. No postoperative concerns were raised during this time with aspermia achieved on subsequent semen analysis for all cases.

Discussion

This scoping review identifies a paucity of evidence surrounding laparoscopic vasectomy for desired male infertility as part of family planning. In particular, only eight cases have been reported in the literature from the 1990s and early 2000s, with no reported cases in the past two decades. Despite the scarcity of evidence, our scoping review highlights that laparoscopic vasectomy can be performed safely and with good efficacy.

When evaluating individual studies included in this review, it is evident that laparoscopic vasectomy has a unique niche where it is considered appropriate from a resource, risk, and cost perspective. Specifically, all cases of laparoscopic vasectomy identified were performed in the context of concomitant pathology that required synchronous laparoscopic management. In this way, the barriers of laparoscopic instrument cost, surgical expertise, and anaesthetic duration are mitigated by the necessity of laparoscopic management of the alternate pathology. Furthermore, for patients already undergoing intra-abdominal laparoscopy, the additional scrotal incisions confer further risk of local complications including haematoma, infection, swelling, and postoperative scrotal discomfort [8]. These have been estimated to occur on the order of 1-6% [8]. It is apparent from the patient perspective that this risk may be unjustified, with patients consenting specifically for laparoscopic vasectomy in these instances as a means of risk prevention [6,12]. Moreover, laparoscopic vasectomy in these cases may also be considered the least invasive means, with an estimated additional five minutes of operative time compared to a likely longer duration of anaesthesia required to either reprepare and drape the already anaesthetised patient for scrotal surgery or for rebooking the patient for a second standalone traditional vasectomy [11]. Therefore, in preoperative settings where a patient is being considered for elective vasectomy and elective laparoscopy for an indication such as repair of an inguinal hernia, synchronous laparoscopic vasectomy may be considered an efficient approach in management plans. Analogous approaches are similarly adopted in other fields, including in gynaecology where clinicians may opt to perform opportunistic laparoscopic tubal ligations in the context of concurrent lower uterine segment caesarean section. This, however, affirms that standalone laparoscopic vasectomy is a highly impractical approach to male surgical contraception [6].

When considering additional limitations of laparoscopic vasectomy, a clear consideration is the difficulty of vasectomy reversal. With scrotal vasectomy, division of the vas deferens within the scrotum allows the possibility of return to fertility with reversal via a scrotal or inguinal vasovasostomy [14,15]. With laparoscopic vasectomy, the possibility of reversal and return to fertility is markedly lower as the vas deferens is divided within the pelvis. Previously, it was generally considered not to be possible, given that there had been no documented cases of retroperitoneal vasovasostomy due to a lack of technology to allow reconstruction of the retroperitoneal pelvic vas deferens. With the advent of robot-assisted surgery, intra-abdominal and retroperitoneal vasectomy reversal has become a possibility; however, the opportunities for this are limited due to surgical expertise and resource availability [16]. Therefore, for patients where laparoscopic vasectomy is considered, judicious counselling regarding the irreversibility of this procedure should take place. Furthermore, clinicians may consider also discussing alternative options for fertility, such as assisted-reproductive technologies including in vitro fertilisation (IVF) or artificial insemination. Moreover, in cases of emergent laparoscopy, such as for infectious aetiology or in settings of surgical oncology, there remains a theoretical risk of contamination or tumour seeding. The evidence at this stage does not support laparoscopic vasectomy for these indications. Lastly, surgical preference and expertise must also be considered. Laparoscopic vasectomy, as demonstrated in this review, is performed only in rare instances with a significant paucity of evidence within the last two decades. Given the rarity of the procedure, there are potential risks that may be conferred to the patient if the laparoscopic approach is undertaken by inexperienced surgeons. In particular, there is a risk of damage to nearby structures during laparoscopy such as bowel, spermatic cord structure and inferior epigastric vessels, which are less at risk during routine scrotal vasectomy. Therefore, if laparoscopic vasectomy were to become a more common practice, there is a need for surgeons to further upskill in this competency.

To the authors’ knowledge, this is the first review to systematically explore outcomes related to laparoscopic vasectomies in humans. However, when evaluating the evidence on laparoscopic vasectomy, there are notable limitations to consider. Specifically, the findings of this scoping review include highly heterogeneous case reports of small sample size. Additionally, the follow-up period of the reported cases from the included studies was highly variable and short where data was available. For vasectomy patients, follow-up at the three-month mark with semen analysis is generally considered adequate. Our review, however, demonstrated follow-up periods of one week and one month in two separate studies, which are insufficient to confirm efficacy. Furthermore, there was a noticeable lack of reporting of follow-up outcomes, in particular efficacy as measured by aspermia. Overall, the safety and efficacy of laparoscopic vasectomy remain poorly characterised with a lack of best-practice principles to guide the development of a standardised approach to patient selection. There is a need for further prospective trials with larger sample sizes and longer follow-up periods to better characterise these outcomes.

## Conclusions

Laparoscopic vasectomy is a valid option for individuals undergoing synchronous laparoscopy for the management of concurrent pathology. Currently, the evidence base supporting the real-world practicality of this, however, is limited, due to a small number of select cases and short duration of follow-up. Prospective clinical trials of larger sample size are required to evaluate the effectiveness of laparoscopic vasectomy.
